# Successive *Acanthamoeba* Corneal Isolates Identified in Poland Monitored in Terms of In Vitro Dynamics

**DOI:** 10.3390/microorganisms11051174

**Published:** 2023-04-29

**Authors:** Lidia Chomicz, Jacek P. Szaflik, Beata Szostakowska, Justyna Izdebska, Wanda Baltaza, Monika Łazicka-Gałecka, Agnieszka Kuligowska, Anna Machalińska, Paweł J. Zawadzki, Jerzy Szaflik

**Affiliations:** 1 Department of Medical Biology, Medical University of Warsaw, 00-575 Warsaw, Poland; 2 Department of Ophthalmology, Independent Public Clinical Ophthalmology Hospital, Medical University of Warsaw, 00-576 Warsaw, Poland; 3 Department of Tropical Parasitology, Faculty of Health Sciences, Medical University of Gdansk, 80-210 Gdańsk, Poland; 4 Department of Public Health, Medical University of Warsaw, 02-097 Warsaw, Poland; 5 First Department of Ophthalmology, Pomeranian Medical University, 70-111 Szczecin, Poland; 6 Clinic of Cranio-Maxillo-Facial and Oral Surgery and Implantology, Medical University of Warsaw, 02-005 Warsaw, Poland; 7 Laser Eye Microsurgery Centre Clinic of Prof. Jerzy Szaflik, Brand Med Medical Research Centre, 00-215 Warsaw, Poland

**Keywords:** *Acanthamoeba* keratitis, corneal strain diagnostics, confocal microscopy, molecular techniques, monitoring in vivo/in vitro, dynamics of amoeba forms

## Abstract

Background: Amoebae of the genus *Acanthamoeba* cause a sight-threatening infection called *Acanthamoeba* keratitis. It is considered a rare disease in humans but poses an increasing threat to public health worldwide, including in Poland. We present successive isolates from serious keratitis preliminary examined in terms of the identification and monitoring of, among others, the in vitro dynamics of the detected strains. Methods: Clinical and combined laboratory methods were applied; causative agents of the keratitis were identified at the cellular and molecular levels; isolates were cultivated in an axenic liquid medium and regularly monitored. Results: In a phase-contrast microscope, *Acanthamoeba* sp. cysts and live trophozoites from corneal samples and in vitro cultures were assessed on the cellular level. Some isolates that were tested at the molecular level were found to correspond to *A. mauritanensis*, *A. culbertsoni*, *A. castellanii*, genotype T4. There was variability in the amoebic strain dynamics; high viability was expressed as trofozoites’ long duration ability to intense multiply. Conclusions: Some strains from keratitis under diagnosis verification and dynamics assessment showed enough adaptive capability to grow in an axenic medium, allowing them to exhibit significant thermal tolerance. In vitro monitoring that was suitable for verifying in vivo examinations, in particular, was useful to detect the strong viability and pathogenic potential of successive *Acanthamoeba* strains with a long duration of high dynamics.

## 1. Introduction

The primary free-living acanthamoebae (FLA) distributed in natural and man-made environments exist in two forms: as the active vegetative trophozoites with characteristic protrusions—acanthopodia—and as double-walled dormant cysts developing after the growth phase and in harsh conditions. The amoebae occur in soil, air, dust, on/in vegetables, in animals, and in different aquatic habitats; they survive in domestic tap water, municipal sewage systems, in chlorinated swimming pools, and in air conditioning units [1,2,3,4,5,6,7]. They have also been detected in hospital environments on equipment surfaces, on accessories, in dialyzers, on surgical instruments, in dental irrigation units, and in contact lenses and their boxes. As *Acanthamoeba* strains can enter human tissues and exist as endozoic forms, they are believed to be amphizoic organisms able to exist as free-living forms and as facultative parasites. The amoebae were detected on skin, in paranasal sinuses and lungs, and among oral cavity microbiota associated with gingivitis and periodontitis [8,9,10]. They are known as causative agents of granulomatous amoebic encephalitis (GAE), a rare, usually fatal disease developing in immunocompromised persons, patients under immunosuppressive therapy, and HIV/AIDS patients [2,5,11].

Some *Acanthamoeba* strains generate serious human health threats as the causative agents of vision-threatening *Acanthamoeba* keratitis (AK). The disease occurs mainly in immunocompetent persons; nowadays, contact lens wearers are at the highest risk of this disease [12,13,14,15,16,17,18,19,20,21,22,23,24,25,26]. AK can also occur in persons not using contact lenses: micro-injuries of corneal epithelium, ocular surgery, and exposure of the eye to natural water bodies containing *Acanthamoeba* forms are predisposing circumstances for this ocular amoebic infection in humans [27,28,29,30,31]. Usually, one eye is affected; however, bilateral infections have also been reported. Some *Acanthamoeba* strains, which are potential agents of this ocular disease in humans, are common in natural and man-made environments. AK includes non-specific symptoms similar to those observed in the course of viral, fungal, or bacterial keratitis. In the AK diagnosis, non-invasive in vivo methods are applied [31,32,33,34,35,36,37,38,39,40,41,42,43,44,45,46,47,48]. Examinations of corneal scrapings may directly identify *Acanthamoeba* developmental forms by detection of the live protozoans. The in vitro cultivation of samples acquired from corneal scrapings is useful for the confirmation of the diagnosis of AK and the assessment of morpho-physiological amoebic characteristics. The evaluation of *Acanthamoeba* isolates at the molecular level should be made by PCR and other molecular techniques [18,24,29,30,31,38,39,40,49,50,51,52,53,54,55,56,57].

*Acanthamoeba* keratitis is challenging in terms of differential diagnosis, among others, because the amoebae may transmit endosymbionts that are potentially pathogenic for humans and are able to survive/proliferate intracellularly within the amoebae [32,33,34,35,36,37,38].

The treatment of AK is often unsuccessful due to extremely high resistance of the cysts of *Acanthamoeba* to chemical agents, disinfectants, and drugs [20,38,51].

The literature and our studies emphasize that human AK that easily leads to blindness caused by pathogenic strains of *Acanthamoeba* has recently been diagnosed more often worldwide [12,13,14,15,41,42,43,44,45,46,51,56].

In this interdisciplinary study, we present successive isolates originating from complicated keratitis incidents previously recognized incorrectly and unsuccessfully treated in various units. We examined and evaluated the material to identify etiological agents of this keratitis, assess the usefulness of particular methods for a comparison of the detected strains that are pathogenic for humans, and monitor of their population dynamics.

## 2. Materials and Methods

The study pertains to material originating from incidents of serious eye infections in 12 women aged 22–48 years and 8 men aged 18–70 years who were ineffectively treated in various ophthalmic units. Because of previous unsuccessful treatment of keratitis, there was a need for diagnostic verification. The study was performed in accordance with the tenets of the Declaration of Helsinki. In the search for pathogenic agents of keratitis, clinical symptoms that were visible in slit-lamp and in vivo confocal microscopy, appearing in the early and advanced infection stages, were taken into account.

Laboratory methods of species identification were based on combination of morphological and molecular characterization.

Samples originating from keratitis material that were initially examined using the phase-contrast microscope were assessed in wet-mount slides based on the amoebic morphology.

Ten samples of the material were investigated for specific identification using molecular techniques. DNA extraction from the material was performed using the commercial Sherlock AX Kit (A&ABiotechnology, Gdynia, Poland). The ASA.S1 fragment of *Acanthamoeba* 18S ribosomal RNA gene was amplified using JDP1 (5′GGCCCAGATCGTTTACCGTGAA3′) and JDP2 (5′TCTCACAAGCTGCTAGGGAGTCA3′) primers targeting ~450 bp fragment of the gene [57]. The reaction mixture consisted of 12.5 μL PCR Master MixPlus High GC (ready-to-use PCR mixture containing Taq DNA polymerase, PCR buffer, MgCl_2_, and dNTPs; A&A Biotechnology), 1 μL each primer (concentration 10 μm), and 2.5 μL DNA template, supplemented with deionized water up to 25 μL. Amplifications were performed in a GeneAmp PCR System 9700 thermocycler (Applied Biosystems, Waltham, MA, USA) according to following protocol: 3 min at 95 °C (initial denaturation) followed by 35 cycles of denaturation at 95 °C for 60 s, annealing of primers at 60 °C for 60 s, strand elongation at 72 °C for 3 min, and a final extension step of 10 min at 72 °C. PCR products were analyzed using the GelDoc-It Imaging Systems (UVP, Upland, CA, USA) after electrophoresis on agarose gel (Sigma, St. Louis, MO, USA), stained with Midori Green DNA Stain (Nippon Genetics Europe, Duren, Germany). Direct sequencing was performed using standard procedures and amplification primers; obtained sequences were analyzed using GeneStudio™ Professional (Thermo Fisher Scientific, Waltham, MA, USA) and compared with the sequences available in the GenBank using NCBI BLAST (identification of *Acanthamoeba* isolates to species level) (http://www.ncbi.nlm.nih.gov/BLAST (accessed on 1 September 2021–30 January 2023).

Simultaneously, to assess in vitro dynamics of particular strain, the cultivation of the samples was performed under axenic conditions in the absence of external live food organisms, as in our earlier studies [31,39,50,58,59]. Cultures were grown in vitro in sterile 15 mL tubes with the liquid medium composed of Bacto Casitone, Difco (BSC) dissolved in water enriched with 10% calf serum, with addition of aqueous solution of antibiotics, namely streptomycin and penicilin, at 24 °C and sub-cultured into this medium twice a month. The dynamics of *Acanthamoeba* strains parallel cultivated were in vitro monitored and compared to one another. Changes in overall numbers of amoebae, the ability of trophozoites to multiply, and trophozoite and cyst proportions were directly counted with aid of the Bürker hemocytometer. Ranges of three counts of amoebae calculated for 1 mL of the medium were compared for particular strains and assays. Samples of several amoeba strains were also exposed to 37 °C (near human eye temperature) to test a tolerance to changes in temperature on 5th day after a sub-culturing and monitored from 7th day 3–5 times a week in the exponential growth phase during in vitro cultivation. Dynamics of amoebic strains were monitored during each sub-culturing for 7 days in the logarithmic growth phase. Results were analyzed statistically (ANOVA, Student–Newman–Keuls method; the level of statistical significance was set at *p* < 0.05).

During laboratory differential diagnosis, samples obtained from corneal material were also routinely tested with microbiological techniques. Preliminary identification of Gram-positive and Gram-negative bacteria strains and conventional in vitro techniques were used for detection of fungi and bacteria.

## 3. Results

The material assessed in our study originated from 20 keratitis cases in which one of the two eyes was affected. In 14 of the incidents (70%), antibacterial or/and antifungal medications were unsuccessfully applied in other units; thus, misdiagnoses were taken into account by us. Investigations were performed to identify the etiological factors of keratitis to verify diagnoses.

Wearing contact lenses (CL), which is a predisposing risk factor for amoebic eye infections, was documented in 17 cases (85%). Among these cases, several were associated with washing CL in tap water and showering and/or swimming in a pool with CL on. In the patients who were not wearing contact lenses, swimming in lakes and swimming in pools were probable risk factors.

There were different durations as well as intensities of clinical symptoms in 18 keratitis cases—ranging from 5 to 38 days—until proper diagnosis and various times when the anti-*Acanthamoeba* therapy was started. In two other misdiagnosed keratitis cases, the affected eyes were treated unsuccessfully over six months. Drug-combined therapy involved mainly chlorhexidine digluconate, polyhexamethylene biguanide PHMB, and propamidine isethionate Brolene with an addition of antibiotics; steroids were also used. In some cases of AK, surgical interventions, deep anterior lamellar keratoplasty (DALK) or penetrating keratoplasty, amniotic membrane transplantations, or cataract surgery were needed.

In the clinical picture of the keratitis incidents included in our study, photophobia, reduced visual acuity, lid edema, and redness appeared in all cases with different intensities. Symptoms that were visible in slit-lamp and in vivo confocal microscopy, appearing in the early and advanced infection stages, were taken into account. The hyperreflective tissues, epithelial inflammation, size and depth of stromal infiltration or ulcer, and presence or absence of hypopyon were assessed by non-invasive in vivo tests in the slit-lamp biomicroscopy. The occurrence of unbearable pain, epithelial diffuse edema progress to dendriform ulcer, and characteristic ring-shaped infiltration successively involving deep stromal layers, which were considered as factors indicating *Acanthamoeba* keratitis, were found in 12 incidents during the monitoring of clinical pictures.

**Figure 1:**
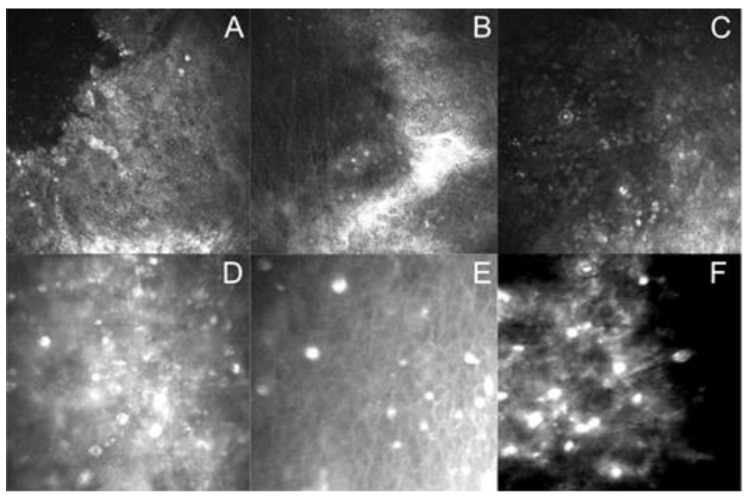
Representative in vivo confocal microscopy of corneal epithelial layer showing the presence of hyper-reflective objects—double-walled *Acanthamoeba* cysts scattered or/and arranged in characteristic chains. (**A**–**C**) HRT3 RCM images (Heidelberg Engineering, Heidelberg, Germany), (**D**–**F**) ConfoScan4TM images (Nidek Technologies, Padua, Italy).

In vivo confocal microscopy was applied in 14 cases for the assessment/verification of the diagnosis. The presence of hyper-reflective objects scattered or arranged in characteristic chains—double-walled, polygonal, or round *Acanthamoeba* cysts, with their outer wall more reflective than the internal walls—was detected by this technique. The visualization of hyper-reflective objects was positive in 16 incidents (80%). However, there were various effects of monitoring related to the previous lack of progress of therapy; in the majority of incidents, hyper-reflective objects, namely amoeba cysts, were detected in the confocal microscopy three weeks after the first keratitis symptoms appeared. The duration of the severe keratitis symptoms was longer, and proper diagnosis was delayed, ranging from 21 to 38 days. Representative confocal microscopy images of corneal layers showed the presence of the hyper-reflective objects; the double-walled *Acanthamoeba* cysts are presented in Figure 1.

### 3.1. Laboratory Differential Diagnosis

During the differential diagnosis, the material obtained from affected eyes was tested in the parasitological laboratory for confirmation of *Acanthamoeba* keratitis. Initially, during the light microscopy examinations, samples of scrapings were directly examined in wet-mount slides using a phase-contrast light microscope (100× and 400×) to visualize cysts or/and trophozoites and assess amoebic strains at the cellular level. Isolated amoebae were identified at the group level (*Acanthamoeba* spp., group I–III), according to Pussard and Pons (1977) [60], mainly based on size, cyst morphology, and the number of opercula. The most commonly detected amoebae corneal isolates were identified and classified as belonging to *Acanthamoeba* sp., group II: live trophozoites (14–38 μm), with pseudopodia and characteristic protrusions, acanthopodia with a nucleus and a prominent centrally placed nucleolus, cysts (7–24 μm) with their two cyst walls wrinkled ectocyst, and a polygonal or round-to-ovoid endocyst.

The results of 10 corneal isolates examined at the molecular level revealed 99.1–100% homology in the obtained sequences, with those available in the GenBank identifying particular isolates as belonging to the T4 genotypes. The material was derived from AK cases: eight contact lens wearers and two non-contact-lens wearers. The obtained sequences are deposited in GenBank under accession numbers MZ401143–MZ401152; the cultured subsequent isolates were in line with *A. mauritanensis*, *A. culbertsoni*, and *A. castellanii*, genotype T4. These results were in line with those from the corneal isolates that were identified on the cellular level as species belonging to the *Acanthamoeba* genus.

A compilation of data on the material investigated for the confirmation of *Acanthamoeba* keratitis (AK) is presented in Table 1.

#### Assessment of In Vitro Cultivation

**Figure 2:**
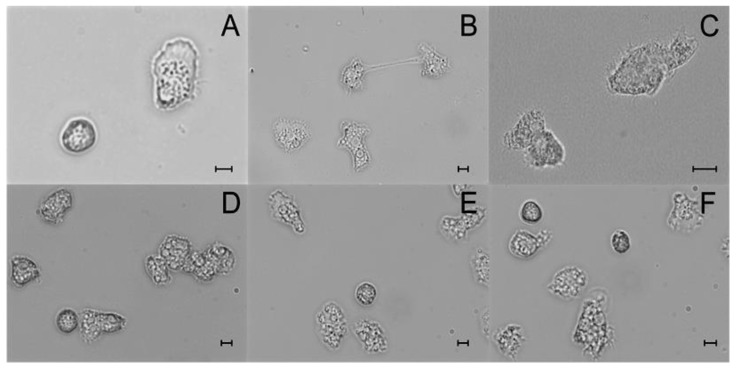
Light micrographs of live *Acanthamoeba* trophozoites and cysts in wet-mount slides from axenically cultured corneal isolates. (**A**), 9m *A. castellanii* trophozoite and double-walled cyst; (**B**), 8m dividing *A.castellanii* trophozoite; (**C**), 5f trophozoites *A.mauritanensis* with numerous acanthopodia; (**D**), 1f *A.polyphaga* trophozoites with numerous digestive vacuoles; (**E**,**F**) *Acanthamoeba* sp. cysts and trophozoites; note characteristic acanthopodia and double-walled cysts. Scale bars = 10 µm.

The cultures were maintained as long as *Acanthamoeba* corneal strains survived. In vitro cultured corneal samples were examined with a phase-contrast light microscope as the direct scraping material. Regular monitoring confirmed AK for 12 cases by the detection of live amoebae. The *Acanthamoeba* infections were confirmed based on the morpho-physiology of the developmental stages. The trophozoites (~18–40 μm) moving by acanthopodia with spine-like protrusions and the double-walled cysts of *Acanthamoeba* (~10–20 μm) were revealed. Light micrographs of live *Acanthamoeba* trophozoites and cysts in the wet-mount slides from cultured corneal isolates are presented in Figure 2.

A comparative assessment of the cultures showed changes in protozoan density that appeared with varying intensity in particular amoebic populations. A low overall number of amoebae was detected in the cultures of some strains with short symptom duration before proper diagnosis.

*Acanthamoeba* corneal isolate dynamics were evaluated in regard to in vitro viability of the particular strains and were expressed as, among others, the trophozoites’ ability to intensely multiply, the overall amoeba number, and the duration of the survival time of the cultivated amoebae. For example, the lowest number of amoebae, 12.0–28.0 × 10^3^ (in the range of three counts), detected in the log growth phase was revealed for the weak corneal strain that indicated a short survival time of about one cycle of sub-culturing. For comparison, a distinctly higher number of amoebae was revealed in the corneal strains, which indicated high in vitro activity during 6 or 24 cycles of sub-culturing, in the range of amoeba numbers 24.0–60.0 × 10^3^, 54.3–80.0 × 10^3^, respectively (the level of statistical significance was set at *p* < 0.05).

A comparison of the in vitro dynamics of particular *Acanthamoeba* strains cultured in BSC medium is presented in Table 2.

The population density of subsequent *Acanthamoeba* isolates with the long-time cultivation (3.5 months and more) transferred to 37 °C and monitored during many cycles of sub-culturing was compared with the effect obtained for samples of the strain that were surviving in a culture medium during only one cycle of sub-culturing; there was a statistically significantly higher overall number of amoebae cells revealed in strains after exposure to 37 °C in comparison with their density in 24 °C. Simultaneously, some reduction in *Acanthamoeba* population density was found in the strain, with short survival times in cultures exposed to changed, higher temperatures.

The representative effects of *Acanthamoeba* T4 corneal strains to changed temperatures in the exponential growth phase of the cultivation are presented in Table 3.

The microbiological examinations of scrapings from corneas performed for the verification of initial diagnoses revealed concomitant infections in 10 of 20 AK incidents. The Gram-positive strain *Enterococcus faecalis*, the Gram-negative *Pantoea agglomerans*, *Enterobacter cloacae*, *Pseudomonas aeruginosa*, and fungal co-infections with *Fusarium* sp. and *Candida* spp. were detected. In this interdisciplinary study, the data on infections with *Acanthamoeba* spp. alone as well as the etiologically mixed keratitis, namely *Acanthamoeba* spp. and concomitant fungal and/or bacterial infections, have been included.

## 4. Discussion

The progressive, devastating, sight-threatening *Acanthamoeba* keratitis is still considered a rare eye disease but is an emerging public health threat worldwide, including in Poland; during the last decades, incidents of human infections caused by pathogenic *Acanthamoeba* strains have been detected and reported in a constantly increasing frequency [24,26,30,31,38,39,40,54,55,61,62,63,64].

The cases included in the study presented challenges in terms of AK. There was a wide range of initial non-specific, confusing clinical symptoms, the incorrect identification of pathogenic factors, and ambiguous initial diagnoses or misdiagnoses influencing the prolonged and severe course of this eye disease.

Although a standardized protocol is missing in AK management, it is emphasized that the clinical picture alone is not sufficient to identify the etiological agent of amoebic keratitis in humans [38,39,48]. Clinical manifestations of AK are similar to those appearing in viral, bacterial, or fungal keratitis. Moreover, the amoebae are known as vehicles/reservoirs/sources of various bacteria, fungi, viruses, and protists, which can survive and multiply within the amphizoic amoebae [32,33,34,35,36,37]. These microorganisms may be causative agents of concomitant/secondary/mixed corneal infections.

In the present study, mistakes in the initial identification of the infectious causative agents of keratitis were found in most cases. It is noteworthy that while AK was finally shown in all 20 incidents, concomitant infections with bacteria and/or fungi that are pathogenic for humans were revealed in 50% of the analyzed cases. It was not possible to assess if the microorganisms were endogenous or exogenous in origin; however, their influence on the difficulties of the correct assessment of corneal isolates under suspicion with AK cannot be excluded.

Diagnostic approaches for AK include methods with different sensitivities: in vivo confocal microscopy, direct microscopic examinations of corneal material, in vitro cultures useful for the detection of causative factors of keratitis, and PCR-based techniques. It is considered that the direct detection of a causative agent in a cornel scrape specimen is the only reliable diagnostic method for AK; culture remains the gold standard of the *Acanthamoeba* laboratory diagnosis, and several PCR-based techniques have been well-established and have increased sensitivity significantly [38,52,57,64].

In this study, the direct detection of the causative agents—amoeba developmental forms—was very important for the proper diagnosis as well as for the verification of common misdiagnoses. A comparison of characteristics of the successively detected isolates identified on the cellular level as *Acanthamoeba* sp. showed the suitability of contrast phase microscopy for the direct visualization of trophozoites and cysts derived from both corneal isolate material and from in vitro cultures, particularly for cases with a long duration of symptoms until proper diagnosis.

Molecular examinations of cultured samples deriving from ten corneal isolates revealed 99.1–100% homology obtained sequences with those available in the GenBank—identifying the individual isolates to species level, e.g., *A. mauritanensis*, *A. culbertsoni*, and *A. castellanii* belonging to the T4 genotype. The results of molecular tests were in line with the effect of a cellular-level direct microscopic examination of *Acanthamoeba* based on the morpho-physiology of trophozoites and cysts.

It is known that *Acanthamoeba* spp. are able to tolerate various conditions in natural and man-made environments and growth in different culture media, e.g., non-nutrient agar plates seeded with *Escherichia coli*, the most commonly used medium peptone–yeast extract–glucose medium PYG, Bacto Casitone, Difco (Bactocasitone, BSC), and the hardly used PYG–Bactocasitone [38,50,63,64].

In our comparative investigations, we used mainly BSC [31,53,59,62]. We considered the axenic medium with the absence of external live food organisms suitable in this study for in vitro cultivation and for monitoring the subsequent *Acanthamoeba* strains.

In the present study, the evaluation of in vitro cultivation of the *Acanthamoeba* corneal strain populations performed under axenic conditions showed different durations of high in vitro activity of amoebic strains from one cycle of sub-culturing to many months and high viability. The thermo-tolerance detected in the subsequent *Acanthamoeba* isolates cultured in vitro expressed that clear strong population dynamics, higher amoeba density, and the ability to intense multiply at high temperatures corresponded to strong viability and long survival time in the axenic culture medium. The ability shown in this study for subsequent *Acanthamoeba* isolates is considered the indicator of the pathogenic potential of a given isolate [31,38,53].

## 5. Conclusions

Our work, based on data from the verification process of *Acanthamoeba* corneal isolates from complicated AK cases, provides information about the differences in amoeba population density and in the duration and the in vitro ability to intensely multiply. To our best knowledge, this is the first Polish study evaluating the amoeba strains *A. mauritanensis* and *A. culbertsoni* in terms of their possibility to grow in vitro that resulted in the confirmation of long survival time of the amoebae isolates in this cultivation conditions. Furthermore, we demonstrated that some of the *Acanthamoeba* strains under dynamic assessments indicated enough adaptive capability to grow in an axenic culture medium, allowing them to exhibit significant thermal tolerance that is considered an indirect marker of pathogenic strains. The in vitro monitoring was especially useful to reveal variability in the dynamics of these *Acanthamoeba* strains that were obtained from keratitis cases with a long duration of symptoms until proper diagnosis.

The results of the presented study, our experience, and the literature data indicate the various levels of usefulness/value of particular methods of keratitis assessment that depend on, among others, the amoebic population viability and density. Therefore, in complicated incidents that are suspected to be AK, a combined approach involving different methods is highly recommended.

## Figures and Tables

**Table 1:** Compilation of data on material investigated for confirmation of *Acanthamoeba* Keratitis (AK).

Corneal Sample	*Acanthamoeba* Strain	Probable Factors Predisposing to AK	Duration of Symptoms Before Proper Diagnosis	Amoebic Forms Visualized in Contrast Phase Microscopic Slides of:	
				Corneal Scrapings	In vitro Cultures
1f	*A. polyphaga*	swimming in a lake	35 days #	cysts and moving trophozoites	cysts and trophozoites
2f	*A. castellanii*	CL	30 days	cysts	cysts and trophozoites
3f	*A. castellanii*	not identified	24 days	no amoebae detected	a few cysts and trophozoites
4f	*A. castellanii*	CL	5 days	no amoebae detected	cysts and trophozoites
5f	*A. mauritanensis*	CL	35 days #	cysts	multiple cysts and trophozoites
6f	*A. mauritanensis*	CL, washing in tap water	30 days #	cysts and trophozoites	multiple cysts and trophozoites
7f	*Acanthamoeba* sp.	CL	7 days	a few cysts detected	no amoebae detected
8m	*A. castellanii*	CL, swimming in a pool	26 days #	cysts	cysts and trophozoites
9m	*A. castellanii*	CL	38 days #	cysts	cysts and trophozoites
10f	*Acanthamoeba* sp.	CL, eye injuries	6 months #; misdiagnosis	cysts	cysts and trophozoites
11f	*A. culbertsoni*	CL	8 days #	a few cysts detected	cysts and trophozoites
12f	*Acanthamoeba* sp.	CL	10 days #	cysts	cysts and trophozoites
13m	*A. castellanii*	CL	26 days #	multiple cysts and trophozoites	cysts and trophozoites
14m	*Acanthamoeba* sp.	swimming with CL in a pool	10 days #	several cysts	no amoebae detected
15m	*Acanthamoeba* sp.	CL	21 days #	cysts	cysts and trophozoites
16f	*Acanthamoeba* sp.	CL	8 days #	cysts	and trophozoites
17m	*Acanthamoeba* sp.	swimming with CL in a pool	8 days #	no scrapings	not cultivated
18m	*Acanthamoeba* sp.	not identified	6 months #; misdiagnosis	cysts	cysts and trophozoites
19f	*Acanthamoeba* sp.	showering with CL	26 days #	cysts	no amoebae developed
20m	*Acanthamoeba* sp.	CL, swimming in a pool	8 days #	no scrapings	not cultivated

CL-wearing contact lenses; # cysts detected by in vivo confocal microscope.

**Table 2:** Comparison of in vitro dynamics of particular *Acanthamoeba* strains cultured in BSC medium.

Corneal Sample	*Acanthamoeba* Strain/Accession No in GenBank	Duration of Cultures Survival	Ability to Intense Multiply in Culture Medium
1f	*A. polyphaga* MZ401143	35 months, still active	all time of cultivation, still
3f	*A. castellanii* MZ401144	15 days	during 2 cycles of sub-culturing
4f	*A. castellanii* MZ401145	10 days	during 1 cycle of sub-culturing
5f	*A. mauritanensis* MZ401146	25 months, still active	all time of cultivation
8m	*A. castellanii* MZ401150	3 months	during 2 cycles of sub-culturing
9m	*A. castellanii* MZ401151	3.5 months	during 3 cycles of sub-culturing
10f	*Acanthamoeba* sp. MZ401148	6 months, still active	during 10 cycles of sub-culturing
11f	*A. culbertsoni* MZ401149	7 months, still active	all time of cultivation
13m	*A. castellanii* MZ401152	2 months	during 2 cycles of sub-culturing
15m	*Acanthamoeba* sp.	4 weeks	during 2 cycles of sub-culturing
16f	*Acanthamoeba* sp.	5 weeks	during 2 cycles of sub-culturing
18m	*Acanthamoeba* sp.	2.5 months	during 5 cycles of sub-culturing

**Table 3:** Effect of in vitro exposure of *Acanthamoeba* corneal strains from exponential growth phase of the cultivation to changed temperature.

No.	*Acanthamoeba* Strain	Range of Overall *Acanthamoeba* Number (×10^3^) Range of Cysts (%)			AVG	SD	Range of Overall *Acanthamoeba* Number (×10^3^) Range of Cysts (%)			AVG	SD
		**24 °C**					**37 °C**				
1f	*A. polyphaga*	66.75	97.50	114.60	92.95	24.25	**105.00**	**120.80**	**140.65**	**122.15**	**17.86**
		1.20	2.80	3.20	2.40	1.06	2.00	3.00	3.50	2.83	0.76
4f	*A. castellanii*	12.40	15.75	28.12	18.76	8.28	**10.15**	**14.05**	**15.80**	**13.33**	**2.89**
		2.20	6.00	12.20	6.80	5.05	**2.40**	**3.50**	**7.20**	**4.37**	**2.51**
5f	*A. mauritanensis*	52.24	60.55	73.40	62.06	10.66	**61.30**	**79.30**	**105.50**	**82.03**	**22.23**
		5.20	7.00	8.20	6.80	1.51	4.00	6.50	8.00	6.17	2.02
9m	*A. castellanii*	24.05	45.60	62.24	43.96	19.15	**46.50**	**62.40**	**80.82**	**63.24**	**17.18**
		2.30	4.00	6.00	4.10	1.85	3.00	3.80	7.00	4.60	2.12
11f	*A. culbertsoni*	55.50	75.20	88.00	72.90	16.37	**67.00**	**87.78**	**97.80**	**84.19**	**15.71**
		1.80	3.20	5.50	3.50	1.87	3.00	4.70	5.30	4.33	1.19

The range of three counts calculated for 1 mL of culture medium is compared for five strains. The level of statistical significance was set at *p* < 0.05; statistically significant differences in relation to data of 24 °C have been bolded.

## Data Availability

The data are not publicly available due to patient’s privacy and ethical reasons.
